# A Preliminary Exploratory Study on the Application of Gd‐EOB‐DTPA‐Enhanced MRI for Assessing Gallbladder Ejection Fraction in Cholecystolithiasis Patients

**DOI:** 10.1155/cjgh/8851215

**Published:** 2026-01-06

**Authors:** Ruikun Zhang, Boqian Chen, Xiaobing Li, Xuan Zheng, Yang Liu, Renjie Zhang, Qingteng Zeng, Hengyu Tian, Qinghua He, Shenfeng Wu, Yuan Gao, Zhujing Li, Hanqing Lyu, Jialin Liu

**Affiliations:** ^1^ The First Department of Surgery, Shenzhen Traditional Chinese Medicine Hospital, The Fourth Clinical Medical College of Guangzhou University of Chinese Medicine, Shenzhen, Guangdong, China, gzucm.edu.cn; ^2^ Intensive Care Unit, Shenzhen Traditional Chinese Medicine Hospital, Guangzhou University of Chinese Medicine, Shenzhen, Guangdong, China, gzucm.edu.cn; ^3^ The Fourth Clinical Medical College of Guangzhou University of Chinese Medicine, Shenzhen, Guangdong, China, gzucm.edu.cn; ^4^ Medical Imaging Department, Shenzhen Traditional Chinese Medicine Hospital, Guangzhou University of Chinese Medicine, Shenzhen, Guangdong, China, gzucm.edu.cn

**Keywords:** cholecystolithiasis, gallbladder contractile function, Gd-EOB-DTPA-enhanced MRI

## Abstract

**Objective:**

To preliminarily explore the feasibility and clinical implications of using gadoxetic acid disodium (Gd‐EOB‐DTPA)‐enhanced magnetic resonance imaging (MRI) for assessing the gallbladder ejection fraction (GBEF) in patients with cholecystolithiasis.

**Methods:**

This retrospective analysis encompassed 81 patients with gallstones who underwent Gd‐EOB‐DTPA‐enhanced MRI. Gallbladder volume was measured during fasting and at 1 h after a lipid‐rich meal to calculate the GBEF. Two radiologists independently reviewed the images for GBEF, structural anomalies, and biliary patency.

**Results:**

The mean GBEF was 62.29% ± 25.2%. Sixty patients demonstrated a GBEF > 50%, while 21 had a GBEF ≤ 50%. The imaging also facilitated the identification of gallbladder malformations (41/81) and abnormal pancreaticobiliary junctions (20/81). Bile flow into the gallbladder via the cystic duct and into the duodenum was observed in 66 patients.

**Conclusion:**

This exploratory study suggests that Gd‐EOB‐DTPA‐enhanced MRI is a feasible modality for simultaneous anatomical evaluation and functional assessment of the GBEF in cholecystolithiasis patients. It provides a comprehensive visualization of biliary dynamics. However, the findings are preliminary, and further validation against standard modalities with controlled study design is required to establish its accuracy and clinical utility.

## 1. Introduction

Gadoxetic acid disodium (Gd‐EOB‐DTPA), also known as gadolinium ethoxybenzyl diethylenetriamine pentaacetic acid, is a gadolinium‐based hepatobiliary contrast agent, which is selectively taken up by normal hepatocytes and excreted through the biliary system. It is instrumental in the detection and diagnosis of hepatobiliary system diseases [1, 2]. Gd‐EOB‐DTPA‐enhanced magnetic resonance imaging (MRI) is predominantly utilized to evaluate focal liver lesions, and it has been applied in some studies for cholangiography. In addition to depicting anatomical structures of the liver, blood vessels, and biliary tract, Gd‐EOB‐DTPA‐enhanced MRI provides functional insights into both intrahepatic and extrahepatic bile ducts, including conditions such as biliary obstruction and bile leakage [3, 4]. Serving as a noninvasive and nonionizing imaging technique, Gd‐EOB‐DTPA‐enhanced MRI is extensively employed in the diagnosis and assessment of hepatobiliary and pancreatic diseases. However, the efficacy of Gd‐EOB‐DTPA‐enhanced MRI in the evaluation of gallbladder contractile function in patients with gallstones remains insufficiently researched. Thus, in this preliminary, exploratory investigation, we endeavor to explore the application of Gd‐EOB‐DTPA‐enhanced MRI for assessing the gallbladder ejection fraction in cholecystolithiasis patients, aiming to report initial findings on its feasibility and to delineate the potential and limitations of this method.

## 2. Methods

### 2.1. Study Subjects

We enrolled 81 patients diagnosed with cholecystolithiasis at our institution between December 9, 2021, and May 18, 2023, comprising 47 males and 34 females with an age range of 19–64 years (mean age: 40.93 ± 10.96 years). The inclusion criteria for the study were as follows: (1) diagnosis of gallstones confirmed by color Doppler ultrasound; (2) no metabolic disorders; (3) no prior history of abdominal surgery; (4) isolated cholecystolithiasis without concurrent gallbladder polyps, adenomyomatosis, or choledocholithiasis, and no recent episodes of acute cholecystitis; and (5) no known allergies to contrast media.

### 2.2. Research Methods

All patient examinations were conducted on a Siemens Prisma 3.0T MRI system. The contrast medium used was Gd‐EOB‐DTPA, a liver‐specific agent produced by Bayer. Patients were instructed to fast for 12 h prior to the examination. Post intravenous administration of 12‐mL Gd‐EOB‐DTPA, patients were positioned supine on the examination table, aligning the coil’s transverse axis center with the umbilicus. Imaging sequences included coronal, sagittal, and axial T1‐weighted (T1WI) and T2‐weighted (T2WI) scans, as well as T1WI contrast‐enhanced scans covering the arterial phase, portal venous phase, delayed phase, and hepatobiliary phase. The hepatobiliary phase required axial and coronal images to measure three dimensions of the gallbladder. The imaging scope encompassed the liver, gallbladder, pancreas, spleen, stomach, and parts of the small intestine.

Twenty min after scanning, patients were given 250 mL of liquid milk and 2 fried eggs. Imaging was repeated 1 h later in the same positions to capture postprandial abdominal images. Gallbladder volumes were measured before and after the meal to calculate the gallbladder ejection fraction (GBEF). During the hepatobiliary phase of Gd‐EOB‐DTPA‐enhanced MRI, the longest diameter of the gallbladder was measured in the axial plane to obtain the long diameter and width. In the coronal plane, the vertical diameter was measured. These three measurements were multiplied to calculate gallbladder volume 1. Similarly, gallbladder volume 2 was measured 1 h after a fatty meal. The GBEF was calculated using the formula (V1−V2)/V1 × 100%, commonly used in ultrasound. Two physicians with at least primary qualifications obtained these measurements, and the average was taken. Additionally, the quantity and size of gallstones, the patency of the cystic duct, and the entry of the contrast medium into the duodenum during the hepatobiliary phase were documented.

The criteria for assessing GBEF in patients with cholecystolithiasis were as follows: a GBEF greater than 50% was indicative of normal gallbladder function, while a GBEF of 50% or less suggested impaired gallbladder function [5].

### 2.3. MRI

All patient examinations were conducted on a Siemens Prisma 3.0T MRI system. The contrast medium used was Gd‐EOB‐DTPA. Patients were instructed to fast for at least 6 h prior to the examination. We first obtained conventional T2WI and pre‐enhanced T1WI images. Following the intravenous injection of 0.1‐mL Gd‐EOB‐DTPA per kilogram body weight, we acquired dynamic enhanced scans. The hepatobiliary phase images were acquired approximately 20 min postinjection (prefatty meal). Subsequently, patients consumed a fatty meal (250 mL liquid milk and 2 fried eggs). Postprandial hepatobiliary phase images were then acquired 1 h after the meal. Gallbladder volumes were measured on pre‐ and postprandial images. Volume was estimated by multiplying the three largest orthogonal diameters (long axis, short axis, and height) obtained from axial and coronal images. GBEF was calculated as (Fasting volume − Postprandial volume)/Fasting volume × 100%.

### 2.4. Radiological Evaluation

Two experienced abdominal radiologists, with 12 and 14 years of experience, respectively, independently reviewed the radiological images of all patients. The imaging reports were required to detail not only characteristic features of cholelithiasis and findings related to other abdominal organs but also to document changes in gallbladder volume and ejection fraction, any anomalies of the biliary tract, as well as the patency of the cystic duct and duodenal papilla, both pre‐ and postlipid meal ingestion.

## 3. Results

### 3.1. Preprandial Gallbladder Volume Assessment

Assessing the gallbladder volume prior to a lipid‐rich meal is a crucial step in evaluating its contractile function. For this assessment, patients were required to fast before the examination and then consume a high‐fat meal, typically a 250‐mL fat emulsion or an equivalent fat load. This protocol is designed to provoke GBEF, facilitating the accumulation of bile and allowing for the subsequent evaluation of contractility. All participants underwent preprandial gallbladder volume measurement, with the mean fasting gallbladder volume recorded at 23.47 cm^3^.

### 3.2. Assessment of Gallbladder and Biliary Tract Anomalies

**Figure 1:**
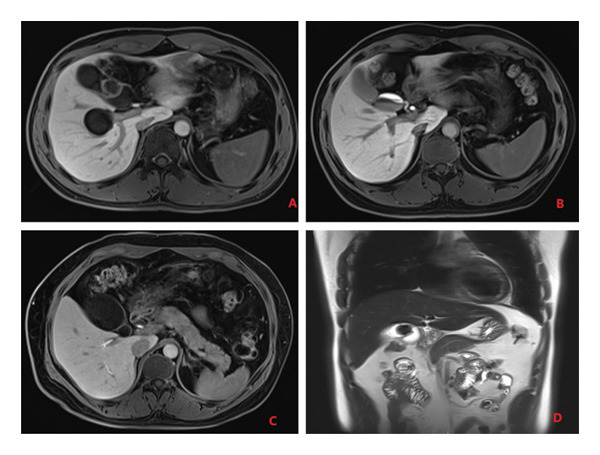
Gallbladder anomaly: (A) septated, (B) gourd shaped, (C) bilobed, and (D) Phrygian cap.

Anomalies in the gallbladder and biliary tract refer to atypical morphological or structural changes in the gallbladder and the connected biliary system. Gd‐EOB‐DTPA‐enhanced MRI offers comprehensive visualization of the gallbladder and biliary tract, enabling the observation of gallbladder shape, the junction between the gallbladder and the common bile duct, and any abnormalities in the pancreaticobiliary junction. A normal gallbladder typically has an oval shape. In this study, out of 81 patients, 41 cases of gallbladder anomalies were identified, including 6 with a Phrygian cap (folded) deformity, 12 with a gourd‐shaped gallbladder, 8 with septation (segregated type), and 15 with a bilobed (double lumen) configuration. Additionally, biliary anomalies were found in 20 patients, primarily presenting as abnormal pancreaticobiliary junctions (refer to Table 1 and Figure 1).

**Table 1:** Anatomical variations of the gallbladder and biliary tract in 81 patients.

Gallbladder/biliary anomaly	Cases (%)
Phrygian cap (folded type)	6 (7.4%)
Gourd shaped	12 (14.8%)
Septated (separated type)	8 (9.9%)
Bilobed (double cavity type)	15 (18.5%)
Abnormal pancreaticobiliary junction	20 (24.7%)

### 3.3. Postprandial Gallbladder Volume Assessment

Assessing the gallbladder volume following a fatty meal is an essential step in evaluating its contractile function. In this assessment, patients’ gallbladder volumes were measured 1 h after consuming a high‐fat load to monitor changes during the postprandial period, thereby evaluating the gallbladder’s ability to contract. This postprandial measurement is instrumental in determining the normalcy of GBEF and identifying potential abnormalities associated with gallbladder diseases. All patients underwent a 1 h GBEF test, with the postprandial average gallbladder volume being 14.64 cm^3^ and the average GBEF reaching 62.39% (Table 2 and Figures 2 and 3). Out of the cases studied, 60 exhibited good GBEF, while 21 showed poor GBEF.

**Table 2:** Gallbladder volume and contraction rate before and after fatty meal in 81 patients.

	**Fasting**	**1 h postprandial**

Gallbladder volume (cm^3^)	23.47 ± 14.40	14.64 ± 8.98
Gallbladder contraction rate (%)	N/A	62.39 ± 20.51

Figure 2Gallbladder images of a 46‐year‐old female patient: (a) in a fasting state and (b) 1 h postprandially following a fatty meal. There is no significant change in gallbladder volume before and after the meal, with a GBEF of 17%, indicating poor gallbladder ejection fraction.

**Figure:**
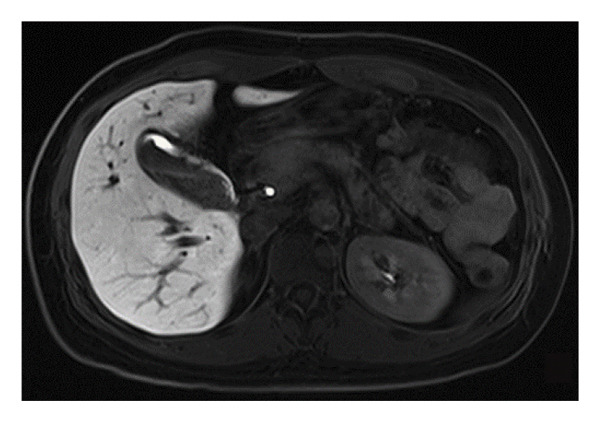
(a)

**Figure:**
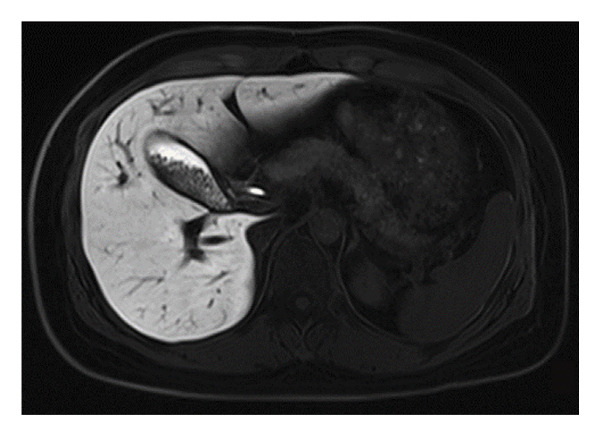
(b)

Figure 3Gallbladder images of a 43‐year‐old male patient: (a) in a fasting state and (b) 1 h postprandially following a fatty meal. There is a marked change in gallbladder volume with a GBEF of 75%, indicating good gallbladder ejection fraction.

**Figure:**
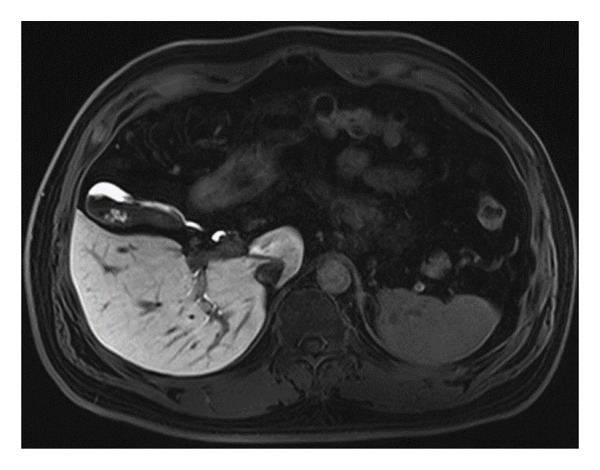
(a)

**Figure:**
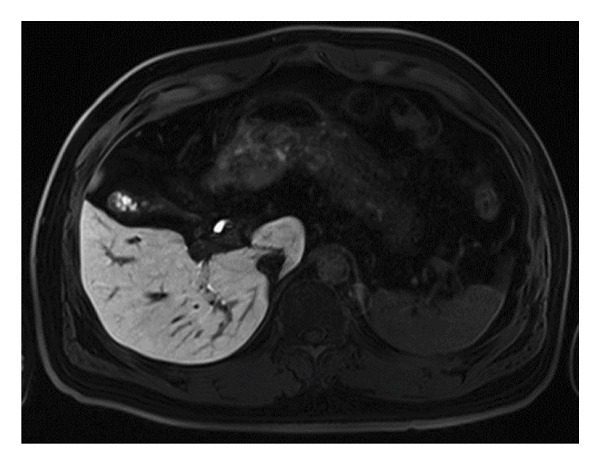
(b)

### 3.4. Evaluation of Bile Entry Into the Gallbladder and Discharge Into the Duodenum During the Hepatobiliary Phase

**Figure 4:**
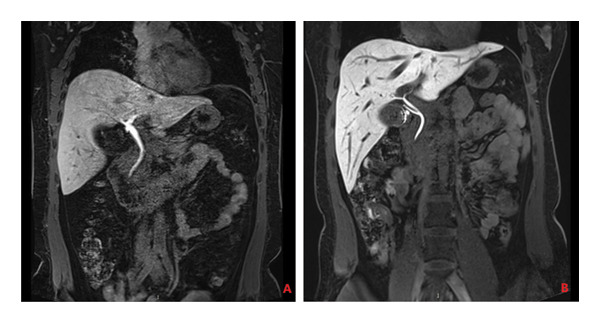
(A) Gd‐EOB‐DTPA‐enhanced MRI hepatobiliary phase image of a fasting 40‐year‐old female patient, showing a turbid cystic duct and impeded bile flow into the gallbladder. (B) Gd‐EOB‐DTPA‐enhanced MRI hepatobiliary phase image of a fasting 36‐year‐old female patient, showing clear bile flow into the gallbladder.

**Figure 5:**
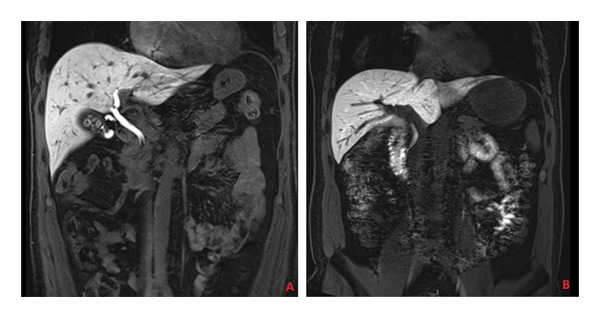
(A) In a 60‐year‐old male patient, fasting Gd‐EOB‐DTPA‐enhanced MRI hepatobiliary phase images reveal an obstructed duodenal papilla, preventing bile from entering the duodenum via the common bile duct. (B) A 50‐year‐old male patient with a patent duodenal papilla, shown on fasting Gd‐EOB‐DTPA‐enhanced MRI hepatobiliary phase images, demonstrates normal bile flow into the duodenum through the common bile duct.

The entry of newly synthesized bile into the gallbladder and its subsequent discharge into the duodenum during the hepatobiliary phase is a critical step in assessing gallbladder function and the bile excretion process. Gadoxetic acid, following uptake by hepatocytes and metabolism through the biliary system, facilitates the dynamic evaluation of bile kinetics. Normally, Gd‐EOB‐DTPA, along with newly produced bile, should enter the gallbladder smoothly, and excess contrast agent should be excreted into the duodenum via the common bile duct. In this study, two radiologists reported that the cystic duct was patent in 66 patients, allowing for the unimpeded entry of bile into the gallbladder. In contrast, 15 patients exhibited impaired bile entry into the gallbladder. Similarly, bile was observed to flow smoothly into the duodenum through the common bile duct in the same 66 patients, whereas 15 patients had difficulties with bile passing into the duodenum (as illustrated in Figures 4 and 5).

## 4. Discussion

This preliminary study demonstrates the feasibility of using Gd‐EOB‐DTPA‐enhanced MRI to derive a GBEF while simultaneously providing a comprehensive anatomical survey of the hepatobiliary system in patients with cholecystolithiasis. Our data, revealing a spectrum of GBEF values and a high incidence of structural anomalies, suggest a potential role for this technique in the integrated assessment of biliary disease. However, these findings must be interpreted as exploratory, highlighting a novel application rather than validating a new standard.

The excretion of Gd‐EOB‐DTPA through the biliary system enables the acquisition of MRI images that offer comprehensive anatomical and functional evaluation of the biliary tree, including the assessment of bile and cystic duct patency. It has been documented that the gallbladder typically displays contrast filling within 20 min after Gd‐EOB‐DTPA administration [6, 7]. In instances of cystic duct obstruction, characteristic MRI findings may include absent or delayed enhancement of the gallbladder. As cystic duct obstruction constitutes a known etiological factor for acute cholecystitis, the application of Gd‐EOB‐DTPA‐enhanced MRI for cystic duct patency evaluation is of considerable diagnostic importance in the clinical setting [8].

The principal finding of our study is that Gd‐EOB‐DTPA‐enhanced MRI can generate a functional parameter—the GBEF—within the framework of a standard morphological examination. This integrative capability represents its most significant potential advantage over existing modalities. Hepatobiliary scintigraphy (HIDA scan), while the functional reference standard, provides limited anatomical detail [9]. Conversely, ultrasound, though excellent for initial anatomical evaluation, has inherent limitations in functional quantification and field‐of‐view [10]. In our cohort, the technique not only quantified GBEF but also provided a clear morphological rationale for functional impairment in a subset of patients. For example, the 15 cases with absent bile entry into the gallbladder or duodenum on imaging would be difficult to differentiate from severe cystic duct dyskinesia or sphincter of Oddi dysfunction on HIDA scan alone. The ability to directly visualize an obstructed cystic duct or an anomalous pancreaticobiliary junction in patients with low GBEF offers a compelling, albeit preliminary, argument for the clinical utility of this comprehensive approach.

The wide range of GBEF values observed (mean: 62.29% ± 25.2%) and the identification of 21 patients with impaired ejection (GBEF ≤ 50%) align with the expected heterogeneity of gallbladder dysfunction in a cholecystolithiasis population. While gallstones can mechanically obstruct outflow or trigger inflammation that impairs gallbladder contractility, it is important to note that reduced GBEF is a nonspecific finding and can be associated with other conditions, including certain medications (e.g., octreotide and calcium channel blockers) [11] and systemic diseases such as Crohn’s disease or diabetes mellitus [12, 13]. It is critical, however, to correctly interpret the physiological meaning of this metric. The GBEF calculated from Gd‐EOB‐DTPA signal intensity reflects the net effect of bile flow into and out of the gallbladder. This net result is governed by multiple factors: hepatocyte function and bile secretion rate, cystic duct patency, gallbladder wall motility, and sphincter of Oddi resistance. Therefore, our use of the term “ejection fraction” is intentional and more accurate than “contractility,” as the latter implies a measure solely of the gallbladder muscle. A low GBEF in this context could stem from true contractile failure, cystic duct obstruction, or even impaired hepatic contrast excretion, a crucial distinction that underscores the need for correlated anatomical imaging.

Gd‐EOB‐DTPA MRI extends beyond assessing GBEF to provide detailed anatomical and functional information about the hepatobiliary system. This includes evaluating the gallbladder wall thickness, identifying gallbladder abnormalities or ectopia, checking the patency of the cystic duct, assessing abnormalities in the pancreaticobiliary junction, and determining if the contrast agent during the hepatobiliary phase has entered the duodenum. In the cholecystolithiasis cases of this study, 41 patients presented with gallbladder malformations, which are known to be a contributing factor to gallstone formation. Although this number seems high, we used strict morphological criteria for assessment. A normal gallbladder is a small, hollow organ, shaped like a pear [14]. When the gallbladder’s morphology deviates from this standard, it may be classified as a malformation. Congenital malformations of the gallbladder include types such as hypoplasia, ectopic gallbladder, and duplication [15]. Additionally, 66 patients had patent cystic ducts and duodenal papillae, allowing the free flow of bile into the gallbladder and duodenum. Gd‐EOB‐DTPA MRI offers a clear and direct visualization of bile flow, confirming whether the bile is entering the cystic duct and duodenum, thereby facilitating the assessment of gallbladder and cystic duct function.

## 5. Limitations

This study has several limitations that must be acknowledged. First, its retrospective design and the lack of a control group or direct comparison with established standards like HIDA scan or ultrasound prevent any definitive conclusions regarding the accuracy and validity of the GBEF measured by Gd‐EOB‐DTPA‐enhanced MRI. Second, the enrollment of consecutive patients was not explicitly documented, and the unusually high prevalence of anatomical anomalies raises the possibility of selection bias, which may limit the generalizability of our findings. Third, the estimation of gallbladder volume using the ellipsoid formula, while practical, is inherently less precise than true 3D segmentation and may introduce error into the GBEF calculation. Fourth, we did not assess interobserver agreement between the two radiologists or test–retest reproducibility, which are crucial for establishing the reliability of the measurement. Finally, the functional interpretation is complex, as GBEF derived from Gd‐EOB‐DTPA signal intensity represents a net effect of bile secretion, cystic duct resistance, gallbladder wall motility, and sphincter of Oddi tone, rather than a pure measure of gallbladder contractility. These limitations underscore the preliminary nature of our work and highlight the need for carefully designed prospective studies in the future.

## 6. Conclusion

This study has provided an initial exploration of the use of Gd‐EOB‐DTPA‐enhanced MRI for assessing GBEF in patients with gallstones. The findings indicate that Gd‐EOB‐DTPA‐enhanced MRI can distinctly delineate the morphological features of the gallbladder and changes in bile dynamics, offering quantitative metrics for GBEF. In conclusion, this study provides preliminary evidence supporting the feasibility of Gd‐EOB‐DTPA‐enhanced MRI for assessing GBEF and providing concomitant anatomical evaluation in cholecystolithiasis patients. The technique shows potential, but our findings are exploratory. Future prospective studies with larger sample sizes, direct comparison to reference standards, and rigorous assessment of measurement reliability are necessary to validate and refine its clinical application.

## Ethics Statement

This research has been approved by the Ethical Committee of Shenzhen Traditional Chinese Medicine Hospital/the Fourth Clinical Medical College of Guangzhou University of Chinese Medicine (approval code: K2021‐006‐05). All subjects signed an informed consent and all methods are done according to the Declaration of Helsinki together with relevant rules and standards.

## Consent

All patients who participated also consented for the publication of this manuscript.

## Disclosure

All authors reviewed and provided approval for the final version of the manuscript.

## Conflicts of Interest

The authors declare no conflicts of interest.

## Author Contributions

Ruikun Zhang, Jialin Liu, and Hanqing Lyu conceived of the study design and determined appropriate surveys and survey items for the study. Ruikun Zhang and Qingteng Zeng collected the data and performed the data analysis. Ruikun Zhang, Zhujing Li, Shenfeng Wu, Boqian Chen, Xiaobing Li, Xuan Zheng,Yang Liu, and Qinghua He interpreted analysis results and participated in drafts and revisions of this manuscript.

Ruikun Zhang and Boqian Chen contributed equally to this work and should be considered as co‐first authors.

## Funding

No funding was received for this research.

## Data Availability

The data that support the findings of this study are available from the corresponding authors upon reasonable request.
